# Supplementary material for: Association of Bone Mineral Density and Coronary Artery Calcification in Patients with Osteopenia and Osteoporosis
Source: Diagnostics (Basel). 2020 Sep 16;10(9):699. doi: 10.3390/diagnostics10090699 (PMC7555969; doi:10.3390/diagnostics10090699)
Supplement: Supplementary file 1 [file diagnostics-10-00699-s001.pdf]

Supplementary Table 1. Distribution of right femoral neck T-score in patients with hypertension or diabetes mellitus

Disease	Right femoral neck			
	**Total**	**Normal** T-score ≥ −1.0	**Osteopenia** −1.0 > T-score > −2.5	**Osteoporosis** T-score ≤ −2.5
Hypertension N (%)	57 (100)	12 (21.1)	37 (64.9)	8 (14.0)
Diabetes mellitus N (%)	21 (100)	5 (23.8)	14 (66.7)	2 (9.5)

Supplementary Table 2. Distribution of left femoral neck T-score in patients with hypertension or diabetes mellitus

Disease	Left femoral neck			
	**Total**	**Normal** T-score ≥ −1.0	**Osteopenia** −1.0 > T-score > −2.5	**Osteoporosis** T-score ≤ −2.5
Hypertension N (%)	57 (100)	16 (28.1)	36 (63.2)	5 (8.8)
Diabetes mellitus N (%)	21 (100)	9 (42.9)	11 (52.4)	1 (4.8)
